# Dedifferentiation-mediated stem cell niche maintenance in early-stage ductal carcinoma in situ progression: insights from a multiscale modeling study

**DOI:** 10.1038/s41419-022-04939-x

**Published:** 2022-05-21

**Authors:** Joseph D. Butner, Prashant Dogra, Caroline Chung, Javier Ruiz-Ramírez, Sara Nizzero, Marija Plodinec, Xiaoxian Li, Ping-Ying Pan, Shu-hsia Chen, Vittorio Cristini, Bulent Ozpolat, George A. Calin, Zhihui Wang

**Affiliations:** 1 Mathematics in Medicine Program, Houston Methodist Research Institute, Houston, TX 77030 USA; 2 Department of Physiology and Biophysics, Weill Cornell Medicine, New York, NY 10065 USA; 3 Department of Radiation Oncology, The University of Texas MD Anderson Cancer Center, Houston, TX 77030 USA; 4 Biozentrum and the Swiss Nanoscience Institute, University of Basel, Basel, 4056 Switzerland; 5 Department of Pathology & Laboratory Medicine, Emory University School of medicine, Atlanta, GA 30322 USA; 6 Immunotherapy Research Center, Houston Methodist Research Institute, Houston, TX 77030 USA; 7 Neal Cancer Center, Houston Methodist Research Institute, Houston, TX 77030 USA; 8 Department of Imaging Physics, University of Texas MD Anderson Cancer Center, Houston, TX 77230 USA; 9 Physiology, Biophysics, and Systems Biology Program, Graduate School of Medical Sciences, Weill Cornell Medicine, New York, NY 10065 USA; 10 Department of Experimental Therapeutics, The University of Texas MD Anderson Cancer Center, Houston, TX 77030 USA; 11 Department of Translational Molecular Pathology, The University of Texas MD Anderson Cancer Center, Houston, TX 77030 USA

**Keywords:** Breast cancer, Translational research

## Abstract

We present a multiscale agent-based model of ductal carcinoma in situ (DCIS) to study how key phenotypic and signaling pathways are involved in the early stages of disease progression. The model includes a phenotypic hierarchy, and key endocrine and paracrine signaling pathways, and simulates cancer ductal growth in a 3D lattice-free domain. In particular, by considering stochastic cell dedifferentiation plasticity, the model allows for study of how dedifferentiation to a more stem-like phenotype plays key roles in the maintenance of cancer stem cell populations and disease progression. Through extensive parameter perturbation studies, we have quantified and ranked how DCIS is sensitive to perturbations in several key mechanisms that are instrumental to early disease development. Our studies reveal that long-term maintenance of multipotent stem-like cell niches within the tumor are dependent on cell dedifferentiation plasticity, and that disease progression will become arrested due to dilution of the multipotent stem-like population in the absence of dedifferentiation. We have identified dedifferentiation rates necessary to maintain biologically relevant multipotent cell populations, and also explored quantitative relationships between dedifferentiation rates and disease progression rates, which may potentially help to optimize the efficacy of emerging anti-cancer stem cell therapeutics.

## Introduction

Female breast cancer diagnoses in the United States exceeded 250,000 for the first time in 2017 (the latest year data available) [1]; up to 25% of these diagnoses are ductal carcinoma in situ (DCIS) [2], the earliest and non-invasive form of breast cancer, representing a major risk factor for invasive cancer [3]. Women who have breast-conserving surgery (lumpectomy) for DCIS have about a 25% to 30% chance of recurrence, but the mechanistic link between DCIS and invasive breast cancer remains largely unclear. Currently, there are no effective diagnostics that are able to accurately predict which patients will experience disease progression beyond stage zero to invasive cancer, and as a result many patients are often exposed to overtreatment and may even receive surgery and radiation therapy [4]. Even the most aggressive estimates predict that only 1 in 3 DCIS patients will progress to invasive disease [5], further highlighting the need for a better understanding of the biophysical mechanisms involved in DCIS.

**Fig. 1:**
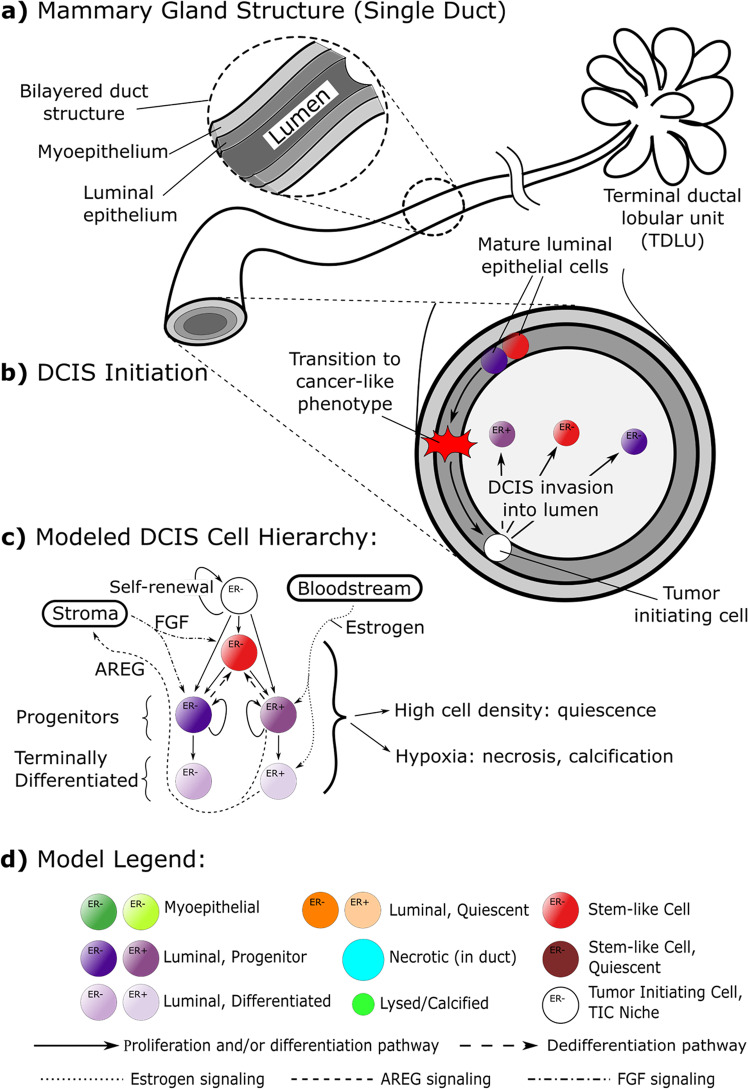
Model schematic and included biology. **a** The mammary gland is composed of bilayered epithelial walls surrounding an inner luminal cavity. This tree-like structure branches away from the nipple into the fat pad, and ducts are terminated by ductal lobular units. **b** The duct wall is composed of outer myoepithelial and inner luminal epithelial cell monolayers. In our model, cancer is initiated by transition of a stem cell within the luminal epithelium into a tumor initiating cell (TIC), which is able to proliferate indefinitely, placing its daughters adjacently into the luminal cavity. **c** Mammary epithelial cells are phenotypically distinct cells and are characterized based on their proliferative potential and the presence or absence of estrogen receptor α (ER + / − , respectively). Proliferation within the DCIS population is regulated by simplified endocrine and paracrine pathways, as shown. **d** Model legend.

DCIS originates from luminal epithelial cells within the mature mammary duct, which undergo unregulated proliferation into the luminal cavity. In the healthy state, bipotent epithelial stem cell niches [6] and a complex interplay of endocrine and paracrine signaling within the mammary gland epithelia are integral to gland homeostasis, where stem cells give rise to daughter cells (daughters) with a proliferative phenotype capable of self-renewal and production of terminally differentiated daughters [7] (Fig. 1). Increasing evidence suggests that tumors originate from cells that gain one or more genetic alterations, resulting in “cells of origin” that initiate cancer and spearhead tumor formation [8]; we refer to these as *tumor initiating cells (TICs)* in this work. These cells of origin must possess the hallmarks of cancer, and the cancer stem cell (CSC) model has emerged to explain these cells of origin [9], which are thought to both maintain a CSC population within the tumor through self-renewal, while also producing daughters through a process of asymmetric division, giving rise to cells that also retain self-renewal capacity but with more differentiated phenotypes [7]. Cell proliferation is regulated by a complex interplay of endocrine and paracrine mechanisms, in part by estrogen-induced stimulation of estrogen receptor positive (ER + ) mammary epithelial cells to upregulate amphiregulin (AREG) production, which stimulates estrogen receptor negative (ER–) cells via fibroblast growth factor (FGF) [10] (Fig. 1c). These pathways are central to cell proliferation in mammary gland development, but may become dysregulated, leading to loss of homeostasis and a transition to a cancer phenotype [11, 12].

Increasing evidence has emerged for the existence of cellular plasticity in differentiation within tumors. In this process, cancer cells may experience phenotypic reversal [9], leading to a transition back to a more stem-like state, and resulting in CSCs that may not have originated from a healthy (non-malignant) stem-like cell [8]. This process is often referred to as dedifferentiation [13], and has been reported in many cancers, including glioblastoma [14], intestinal tumors [15], melanoma [16], and also in breast cancer, where CSCs arise through an epithelial-mesenchymal transition (EMT)-like process facilitated by the ZEB1 promoter [17]. Tumor microenvironmental factors have been shown to play key roles in the dedifferentiation process, including hypoxia [18], Ras-Myc signaling [19], nuclear β-catenin localization and Wnt signaling [20], and TNF-α signaling [16]. These are also thought to play a role in stem cell niche maintenance, where stem cells may also emerge due to dedifferentiation [18, 21]. Specific to breast cancer, dedifferentiation is proposed to be due to ZEB1-based driving of EMT events, which is also required for conversion of non-CSC to CSC and for the maintenance of CSC-like activity [17, 22]. This is also driven by hypoxia [18], oncogenic-induced Yamanaka factors (such as c-Myc or Klf4) [23], or mutated KRAS signaling. This mounting evidence challenges the traditional hierarchy of cellular differentiation, and suggests the need for additional understanding of the role dedifferentiation plasticity plays in cancer [24]. In fact, several groups have made notable strides in quantifying the dynamics of CSC emergence through dedifferentiation events, with a special focus on understanding the prevalence of these events and the conditions under which they may occur [17, 24].

**Fig. 2:**
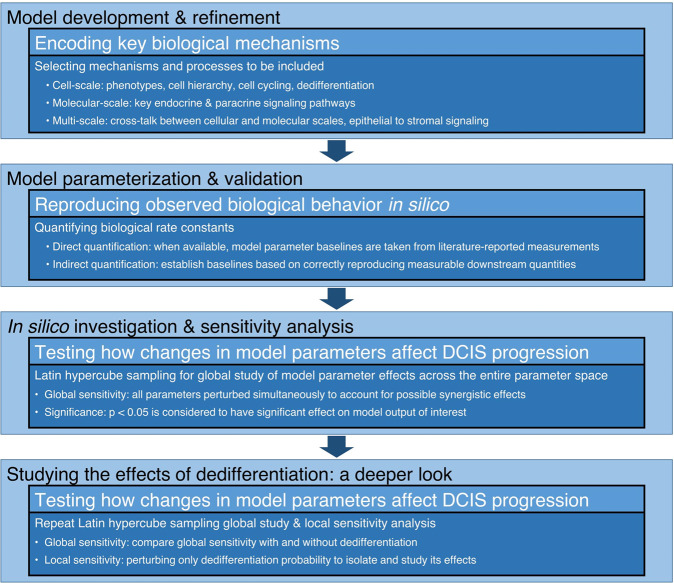
Study flowchart. A graphical description of the study is shown. Briefly, after identifying key biological and physical processes and phenotypic and signaling hierarchies to be included in the model, the model was extensively tested to phenomenologically quantify model parameters that were unavailable in the literature, based on successful reproduction of literature-reported measurements. The full parameter space was examined using Latin hypercube sampling (LHS) parameter perturbation, and paramters with significant effects on model outcomes were identified. Perturbation studies were repeated with and without dedifferentiation, and local and global sensitivity analysis was performed.

Mathematical modeling has yielded significant insights into DCIS development, and into the roles of cellular hierarchies and dedifferentiation in cancer progression; a detailed review is provided in SI. In the work presented herein, we build on our previous modeling studies of the mammary gland and DCIS [25–28] to better understand how cellular dedifferentiation and a combination of other tissue, cellular, and molecular scale factors come together to influence the emergence of the early stages of DCIS (see Table 1), as outlined in (Fig. 2).

**Table 1:** Model parameters perturbed in the global sensitivity analysis.

Parameter	Symbol	Units	Baseline [range]	Distribution	Reference
**Discrete (ABM) parameters**					
Cell cycle time*^,§^	*τ*_P_	hours	20 [16.5 – 23.5]	Uniform	[29, 30]
Symmetric proliferation probability					
-progenitor (is daughter progenitor or differentiated?)*^,†,§§^	*ω*_P_	%	75% [50% – 100%]	Uniform	N/A
-stem (is daughter stem?)*^,†,§^	*ω*_SC_	%	12% [12% ± 6%]	Lognormal	[46]
-stem (are non-stem daughters ER + or ER–?)*^,§§^	*ω*_SC,+/–_	%	50% [36 – 64%]	Uniform	N/A
Apoptosis probability*^,§^	*ω*_Apop_	% Δt^−1^	0.705% [0.10% – 1.31%]	Uniform	[47]
Proliferation cycles before differentiation*^,†,§§^	*P*_max_	Cell cycles	18 [11 – 25]	Uniform	
Cell density threshold for quiescence*^,†,§§^	*θ*_Q_	Cells volume^−1^	70% [50 – 90%]	Uniform	N/A
Dedifferentiation probability^†,§^	*Ω*_DD_	% cell^−1^ cycle^−1^	0.4% [0.1 – 0.7%]	Uniform	[24]
**Hybrid (ABM** ↔ **continuum) parameters**					
Cancer cell oxygen metabolism multiplier (cancer cell oxygen metabolism = λ_C_×*λ*_E_)*^,†, §^	*λ*_C_	Mol cell^-1^ s^−1^	4.5× healthy cell metabolism [3.0 – 6.0×]	Uniform	[48]
Estrogen metabolism*^,§§^	*λ*_E_	Normalized (% baseline)	1.0 [0.9 – 1.1]	Uniform	N/A
FGF uptake/metabolism*^,§§^	*λ*_FGF_	Normalized (% baseline)	0.72 [0.648 – 0.792]	Uniform	N/A
AREG production*^,§§^	*λ*_AREG_	Normalized (% baseline)	0.086 [0.0774 – 0.096]	Uniform	N/A
Estrogen proliferation threshold*^,†,§§^	*θ*_E_	Normalized (% baseline)	0.85 [0.85 ± 0.2]	Lognormal	N/A
FGF proliferation threshold*^,†,§§^	*θ*_FGF_	Normalized (% baseline)	0.7 [0.7 ± 0.15]	Lognormal	N/A

Parameters were tested with either a uniform range [min-max] or a lognormally distributed range [mean ± error factor], with each run under a unique parameter setting determined by LHS. *Parameters perturbed in the full global sensitivity analysis without dedifferentiation. ^†^Parameters perturbed in the dedifferentiation sensitivity analysis. ^§^Parameters quantified from in vivo or in vitro experimental measurements. ^§§^Parameters estimated mathematically or obtained from other modeling work.

## Methods

### Model overview

The basic multiscale agent-based modeling (ABM) framework was published in [27]; interested readers may refer to the SI and other previous publications [25, 28] for descriptions of model design, methods of implementation, and technical details. Briefly, in our model, cells are represented as discrete spherical entities (agents), each with unique phenotypes, positions within the cellular hierarchy, and spatial locations (Fig. 1c, Table 1, Supplementary Table S1), and are free to move lattice-free within the simulated domain (a section of mammary gland duct, Fig. 1c) based on physical interactions with their neighbors and the duct wall. At the molecular scale, continuum molecular profiles (oxygen, estrogen, AREG, and FGF) are described via Fick’s law and solved at each time step using finite element methods. These molecular profiles are then mathematically linked to agents at the discrete scale for explicit feedback between scales, thereby implementing key epithelial and stromal signaling pathways involved in cellular proliferation. Cell agents simulate multiple, sequential proliferation cycles, and may undergo phenotypic transitions according to the cell hierarchy shown in Fig. 1c and determined by the reference criteria and probabilities in Table 1, Supplementary Table S1, and may proliferate, grow, terminally differentiate, enter quiescence, become hypoxic or necrotic, or undergo apoptosis, as determined by the conditions at their physical location (approximated as the agent’s center of mass) and their phenotype. We have made the simplifying assumption that these state transitions (except for growth, which occurs over multiple steps within the time range specified in Table 1) are completed by the start of the next discrete (ABM) time step. Upon mitosis, daughter phenotypes are determined stochastically with probabilities described in Table 1. All simulations presented herein were conducted within a simulated section of mature mammary duct represented as a cylinder 1 mm in length with 200 μm inner luminal duct cavity diameter.

### New model components

#### Stochasticity in cell cycle time

In our previous work, we made the simplifying assumption that all cells would experience equal cell cycle time; that is, the minimum time to complete all cell cycle phases before reaching maturity and being able to undergo another mitosis cycle was held constant. While this allowed for focused study of the effects of variations in cell cycle time on disease progression without the confounding effects of randomness in cell cycle times, it also resulted in an artificial cell cycle synchronicity. In the new version of the model, each daughter cell is assigned a cell cycle time randomized from a bounded lognormal distribution with a mean selected from the range shown in Table 2; means were held constant for each simulation run, but were varied between runs. Baseline mean cell cycle times for mammary epithelial cells were based on cell cycle time of 20 h reported by Shehata et al. [29]. We note that, because we do not explicitly model all phases of the cell cycle for each cell, we imposed a biologically relevant minimum cell cycle time of 16 h based on literature reported values [29, 30] in order to ensure all phases of the cell cycle are complete before another mitosis cycle begins (thereby imposing a lower bound in case a random cell cycle time is selected below this biological threshold).

**Table 2:** Global sensitivity analysis using linear regression without dedifferentiation.

Model output	*R*^2^	Significant model parameters (*p*-value)
Axial advance rate (μm/day)	0.6389	*ω*_P_ (<0.001); *θ*_Q_ (<0.001); *θ*_E_ (<0.001); *θ*_FGF_ (0.001); *ω*_SC_ (0.003); *τ*_P_ (0.005); *P*_max_ (0.015)
Proliferation events per day	0.6599	*θ*_Q_ (<0.001); *ω*_P_ (<0.001); *P*_max_ (0.001); *ω*_SC_ (0.002); *θ*_FGF_ (0.015); *θ*_E_ (<0.024); *τ*_P_ (0.030)
Axial calcification extent (μm)	0.7715	*θ*_Q_ (<0.001); *λ*_C_ (<0.001); *P*_max_ (<0.001); *ω*_P_ (<0.001); *ω*_SC_ (0.036)
Total cells calcified	0.7639	*θ*_Q_ (<0.001); *λ*_C_ (<0.001); *ω*_P_ (<0.001); *P*_max_ (<0.001)
Time to leading edge quiescence (days)	0.2377	*θ*_E_ (0.005); *θ*_FGF_ (0.006); *ω*_P_ (0.012)
Total cell count at arrest	0.7118	*θ*_Q_ (<0.001); *P*_max_ (<0.001); *ω*_P_ (<0.001); *λ*_C_ (0.046);
Total ER + cells at arrest	0.6099	*θ*_E_ (<0.001); *P*_max_ (<0.001); *ω*_P_ (0.001); *θ*_Q_ (0.008); *θ*_FGF_ (0.009); *ω*_SC_ (0.019)
Total ER − cells at arrest	0.6680	*θ*_Q_ (<0.001); *θ*_E_ (<0.001); *λ*_C_ (0.016); *P*_max_ (0.020)

Goodness-of-fit (*R*^2^) of the full multivariate model and parameters found to have significant effects on each model output are shown. All parameters in Table 1 marked with an asterisk (*) were perturbed in a Latin hypercube sampling (LHS) study; parameters not listed here were found to have insignificant effects on model outputs (*p* ≥ 0.05). Progenitor symmetric proliferation probability: *ω*_P_; cell density threshold for quiescence: *θ*_Q_; estrogen proliferation threshold: *θ*_E_; FGF proliferation threshold: *θ*_FGF_; probability of a stem cell having a stem daughter: *ω*_SC_; cell cycle time: *τ*_P_; proliferation cycles before differentiation: *P*_max_; cancer cell oxygen multiplier *λ*_C_.

#### Dedifferentiation

In the work presented here, we have allowed for stochastic dedifferentiation of proliferative phenotype back into the cancer stem cell phenotype. We have also made the simplifying assumptions that quiescent cells do not dedifferentiate, and that terminally differentiated cells do not dedifferentiate back into the proliferative phenotype (we address the implications of this assumption in light of Yamanaka’s discoveries that terminally differentiated cells may revert back to pluripotent stem cells [23] in SI). In addition, cells in our model may not dedifferentiate at the same time they undergo mitosis to avoid introducing artificial variations in the probability distributions for daughter phenotypes (Table 1). Lastly, because we only model a single stem phenotype in our model, agents that have a stem phenotype are unable to dedifferentiate. Agents that meet the above criteria are given the opportunity to stochastically dedifferentiate at each time step such that the dedifferentiation rate per cell cycle is as reported in Table 1, and in each simulation run all agents have the same per-cell-cycle differentiation probability (note that this probability may be set to zero). The dedifferentiation pathway may be manually turned on (*Ω*_DD_ > 0) or off (*Ω*_DD_ = 0) in our simulations, enabling controlled in silico experimentation on the effects of the presence, absence, or stochastic likelihood (*Ω*_DD_) of dedifferentiation events.

### Model analysis

We have previously reported extensive local sensitivity analysis of key model parameters without dedifferentiation [27]. Here, we present an expanded analysis covering a greater number of model parameters in a global analysis [31–39], wherein all model parameters of interest were perturbed simultaneously. For each simulation run, model parameter values were determined via Latin hypercube sampling (LHS), based on values and distributions shown in Table 1. All model simulations were run in serial for 96 wall-clock hours, and model states (including all cell locations, phenotypes, cell volumes, and continuum solutions at all nodes) and outputs of interest were recorded or calculated after every 30 minutes of simulated time. In our first global analysis, many model parameters were perturbed simultaneously without dedifferentiation (that is, dedifferentiation probability *Ω*_DD_ = 0), and multiple regression analysis was conducted to determine parameters that had significant (*p* < 0.05) effects on model outputs of interest. In a second analysis, the dedifferentiation pathway was activated, but parameters tested in the first global analysis that were deemed to be insignificant to all or most of the model outputs of interest were removed from the perturbation analysis (and held constant at their baseline), allowing us to focus on the key parameters deemed to have the greatest influence on DCIS progression. Finally, a third study was conducted wherein only the dedifferentiation probability was perturbed; these studies are outlined in Fig. 2.

## Results

### DCIS progression is ultimately arrested without stem cell replenishment from dedifferentiation: a global sensitivity analysis

**Fig. 3:**
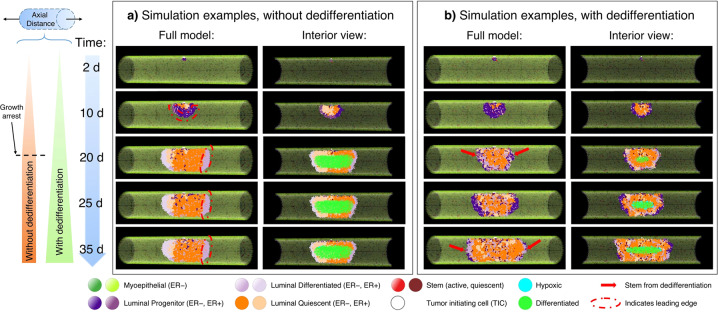
Representative model simulations. **a** Representative model states rendered from a simulation without dedifferentiation are shown as full simulated duct (left) and cross-sectional view (right; cross-section taken along *y* = 0 plane). DCIS is initiated at the top-center of the duct by 5 TIC cells (white) at *t* = 0 days (d); daughters are placed adjacently into the luminal cavity, and DCIS grows away from site of initiation along the leading edge (examples are indicated by red dashed curves). In the example shown, the leading edge has become terminally differentiated at time *t* = 20 days (d), and growth arrest is observed as only minimal further expansion of the DCIS mass occurs after this time. Calcification due to hypoxia is observed (green agents) in the cross-sectional view (right column). **b** Representative model images from a simulation with the dedifferentiation pathway activated. At time *t* = 20 days (d), dedifferentiated cells (lighter purples) are again observed in the leading edge, but in this case new stem cells (red arrows) have emerged in the leading edge; this behavior is again observed in times *t* = 25 days and *t* = 35 days.

**Fig. 4:**
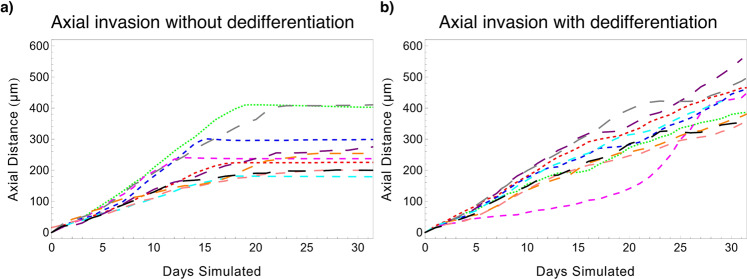
Axial invasion over time from selected simulations. Each simulation run was input with a unique set of parameter values determined by LHS (see Table 1, Supplementary Table S1). **a** Without dedifferentiation, axial invasion is observed to arrest when the stem cell population in the leading edge becomes depleted; as can be seen by curves flattening at higher times. **b** Growth arrest is not seen when the dedifferentiation pathway is active. Only a subset (*n* = 10) of simulations are shown in each panel for ease of visibility; matching colors do not indicate a relationship between curves in each panel.

In a first step, a global sensitivity analysis was performed varying all 13 model parameters marked with an asterisk (*) in Table 1, but without dedifferentiation (that is, dedifferentiation probability *Ω*_DD_ = 0). It was observed that stem cells experienced exponential dilution over time in the absence of dedifferentiation events (an example is shown in Supplementary Fig. S5), often leading to a lack of stem cells in the leading edge. When combined with the finite limit on mitosis cycles imposed on the progenitor phenotype before terminal differentiation, it was observed that the leading edge would ultimately become terminally differentiated in these cases, at which time DCIS progression was arrested (Figs. 3a and 4a). Times to leading edge arrest ranged between 9.1-90.5 days, with an average of 25.9 days. In this study, we truncated model outputs at the time of growth arrest, and results herein represent the state of the model at that time.

Model outputs examined in this study were (1) DCIS axial advance rate per day, (2) average proliferation events per day, (3) axial extent of calcification and time of growth arrest, (4) total cells calcified at time of growth arrest, (5) time before the DCIS leading edge became quiescent, (6) total cell count at time of growth arrest, and (7) total numbers of ER + / − cells at time of growth arrest. A multivariate linear model was constructed for each output of interest, *R*^2^ assessment of goodness-of-fit was performed, and model parameters that showed significant effects (*p* < 0.05 was considered significant) on model outputs of interest were identified. We note that because DCIS advances in both directions along the duct axis (away from the location of tumor initiation), total advance per day and axial extent measures represent the summed change in axial distance in both directions (see Fig. 3).

Multivariate linear regression analysis revealed that only 8 model parameters showed a significant effect on model outputs; these are detailed in Table 2. Of these, progenitor symmetric proliferation probability (*ω*_P_), cell density threshold for quiescence (*θ*_Q_), and proliferation cycles before terminal differentiation in the progenitor phenotype (*P*_max_) were most often significant (in 7/8 model outputs examined), followed by estrogen proliferation threshold (*θ*_E_) (5/8 model outputs), and then by FGF proliferation threshold (*θ*_FGF_), stem cell symmetric proliferation threshold (*ω*_SC_) (stem vs. progenitor daughters), and cancer cell oxygen consumption multiplier (*λ*_C_) (4/8 model outputs). Interestingly, cell cycle time (*τ*_P_) was found to be significant in only 2/8 model outputs examined. The absolute values of the standardized (unitless) effects of each parameter on each examined output are shown in Supplementary Fig. S1.

#### Inclusion of dedifferentiation overcomes growth arrest: global sensitivity analysis with active dedifferentiation pathway

A second global analysis study was performed wherein the dedifferentiation pathway was activated (thus dedifferentiation probability *Ω*_DD_ > 0) and a reduced parameter set consisting of only those identified to be significant in the previous study plus the dedifferentiation probability were perturbed; these are labeled with a dagger (^†^) in Table 1. Multivariate regression was repeated as described above, and model goodness-of-fit and parameter significance on model outputs of interest were evaluated. We note that cell cycle time was excluded from this study, as it was only significant in 2/8 outputs in our first study. Growth arrest of the DCIS cell mass due to terminal differentiation in the leading edge was not observed in this analysis study (see Figs. 3b, 4b); in all simulations, axial growth and cell population increase were observed until the end of the 96 wall-clock hour simulation runs. Stochastic dedifferentiation events were able to overcome the leading edge growth arrest behavior that was observed in the first analysis study (see the previous section), and growth arrest was not observed in any simulation run with dedifferentiation in effect (these are further examined later). Accordingly, time to leading edge growth arrest was not included as a model output examined in this second study.

It was observed that quiescence density threshold (*θ*_Q_) and progenitor symmetric proliferation probability (*ω*_P_) were most influential (that is, they exhibit significant influence on the largest number of model outputs examined) on the examined model outputs, with significant effects on 6/7 outputs of interest. Cancer cell oxygen multiplier (*λ*_C_) showed a significant effect on 5/7 model outputs, followed by estrogen proliferation threshold (*θ*_E_), showing significant effects on 3/7 model outputs, and then by dedifferentiation probability (*Ω*_DD_), maximum proliferation cycles before differentiation (*P*_max_), and FGF proliferation threshold (*θ*_FGF_), each showing significant effects on 2/7 model outputs examined; details are provided in Table 3. Note that the probability of a stem cell having a stem daughter (*ω*_SC_) was included in this analysis, but was found to be insignificant in all cases. Pareto charts depicting the absolute values of the standardized (unitless) effects of each parameter on each examined output are shown in Supplementary Fig. S2.

**Table 3:** Linear regression analysis of global sensitivity analysis with the dedifferentiation pathway enabled.

Model output	*R*^2^	Significant model parameters (*p*-value)
Axial advance rate (μm/day)	0.6940	*θ*_Q_ (<0.001); *ω*_P_ (<0.001)
Proliferation events per day	0.7017	*θ*_Q_ (<0.001); *ω*_P_ (0.001); *λ*_C_ (0.002)
Axial calcification extent (μm)	0.7356	*λ*_C_ (<0.001); *ω*_P_ (<0.001); *Ω*_DD_ (0.018)
Total cells calcified	0.9026	*λ*_C_ (<0.001); *θ*_Q_ (<0.001); *ω*_P_ (0.003); *P*_max_ (0.020)
Total cell count at end of simulation	0.9012	*λ*_C_ (<0.001); *θ*_Q_ (<0.001); *ω*_P_ (<0.001); *θ*_FGF_ (0.001); *Ω*_DD_ (0.004); *θ*_E_ (0.005)
Total ER + cells at end of simulation	0.8142	*θ*_E_ (<0.001); *θ*_Q_ (0.011); *P*_max_ (0.028)
Total ER − cells at end of simulation	0.8805	*θ*_E_ (<0.001); *λ*_C_ (<0.001); *θ*_Q_ (<0.001); *ω*_P_ (<0.001); *θ*_FGF_ (0.001)

Goodness-of-fit (*R*^2^) of the full multivariate model and parameters found to have significant effects on each model output are shown. All parameters in Table 1 marked with a dagger (^†^) were perturbed in a Latin hypercube sampling study; parameters not listed here were found to not have a significant effect on model outputs (*p* ≥ 0.05). Dedifferentiation probability (per cell cycle): *Ω*_DD_; progenitor symmetric proliferation probability: *ω*_P_; cell density threshold for quiescence: *θ*_Q_; estrogen proliferation threshold: *θ*_E_; FGF proliferation threshold: *θ*_FGF_; proliferation cycles before differentiation: *P*_max_; cancer cell oxygen multiplier *λ*_C_.

#### Dedifferentiation overcomes stem cell dilution and terminal differentiation: effects on stem cell population

We then sought to gain insights into the dynamics of stem cell populations across all global sensitivity analysis simulations, with and without activation of the dedifferentiation pathway. At the time of leading edge arrest in simulations without dedifferentiation, the DCIS stem cell population was found to average 0.55% (range: 0.037–5.5%) without dedifferentiation, well below literature reported values of 4-6% [17, 24]. Conversely, in simulations with the dedifferentiation pathway active, stem cell population was found to have a mean of 3.6% (range: 0.50-9.1%) across all simulations, which is in good agreement with the lower end of the literature-reported range.

The percentage of stem cells resulting from dedifferentiation events was found by multivariate linear regression analysis to be largely dependent on the total cell proliferation rate (*p* < 0.001), with greater proliferation rates (and thus faster DCIS ductal advance rates, *p* < 0.001; and total cell count, *p* < 0.001) significantly associated with lower stem cell density in the final DCIS population. All model outputs examined are plotted against final stem cell count in Supplementary Fig. S3. The relation between stem cell density and progenitor cell phenotypes are also shown in Supplementary Fig. S4a, b; however, because dedifferentiation occurs stochastically, these panels merely restate the trend observed in the total progenitor cell population. Stem cell density was also observed to be reduced when greater numbers of hypoxia-induced necrosis and subsequent cell calcification were observed (*p* < 0.001; not shown).

#### Isolating the effects of dedifferentiation using local sensitivity analysis: a deeper look

**Fig. 5:**
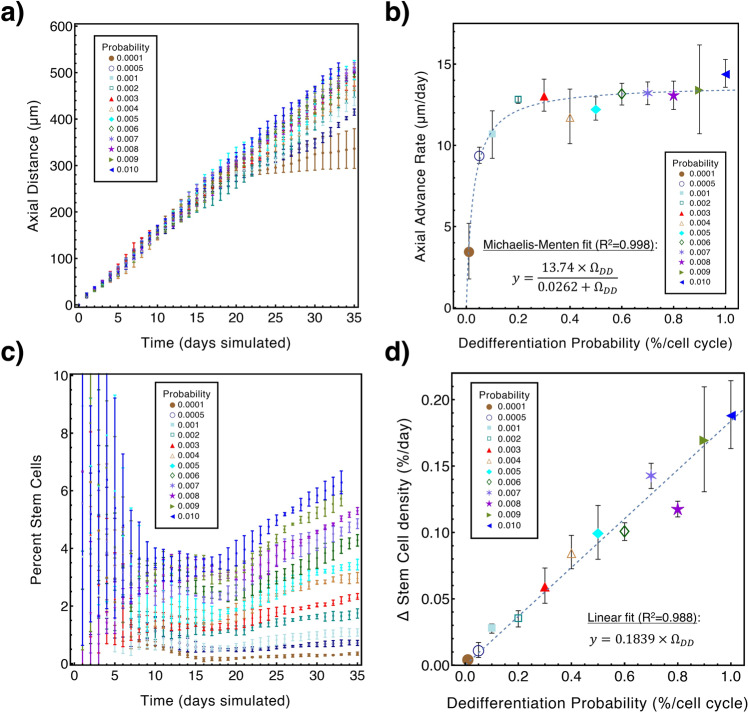
Local sensitivity analysis of dedifferentiation effects on axial advance rate and stem cell population. **a** DCIS axial distance per day shows reduced axial advance at lower dedifferentiation probabilities examined after progenitor mitosis cycle limits are first encountered (*t* = 15 days), while higher dedifferentiation probabilities support consistent advance rates. **b** Average axial advance rate after start of dedifferentiation (*t* ≥ 15 days) was determined via linear regression analysis of data shown in **a**; values are listed in Supplementary Table S2. Michaelis-Menten best fit to data in **b** was found to be axial advance rate = (13.74 *×* *Ω*_DD_)/(0.0262 + *Ω*_DD_) µm/day and is shown (blue, dashed; *R*^2^ = 0.998, *p* < 0.001). **c** Density of stem cells in the total DCIS population; note that at early times, stochastic proliferation results in large percentage variations before steady state is achieved at later times. **d** Linear regression analysis to data shown in C of stem cell density after differentiation events begin (*t* ≥ 15 days; see Supplementary Table S2) reveals a linear relation between dedifferentiation probability and the average daily percent change (Δ) in stem cell density; linear regression revealed average daily change in stem cell percent density = 0.189 *×* *Ω*_DD_ (blue, dashed; *R*^2^ = 0.988, *p* < 0.001). All simulations were performed in triplicate; data shown as mean ± standard deviation (error bars).

In order to gain further insights into the effects of dedifferentiation in DCIS progression, we performed an additional study where only the dedifferentiation probability (*Ω*_DD_) was perturbed while all other parameter values were held at baseline (Table 1, Supplementary Table S1). As an experiment, we expanded the range of dedifferentiation probabilities to 0.01–1.0% in the increments shown in Fig. 5, beyond the more biologically-relevant range listed in Table 1. This range was intentionally imbued with a finer discretization towards the lower end, so that the threshold where dedifferentiation is able to overcome growth arrest or slowing could be identified with higher precision. In this analysis, simulations were not truncated at the time of growth arrest, but instead were truncated at the same time simulated for all tests (*t* = 35 days, later than growth arrest was observed without dedifferentiation (Fig. 4a)). Additionally, our analysis of axial advance rates and rate of change of stem cell density (see Fig. 5) was applied only to data collected after our baseline time for dedifferentiation to occur (that is, *t* > 15 days; Table 2) to focus on steady-state DCIS progression rates. Tests were performed in triplicate for each probability, and model outputs of DCIS axial invasion rate and stem cell percentages were assessed.

DCIS axial advance rate was notably reduced at low dedifferentiation probabilities, but remained consistent at larger dedifferentiation probabilities (Fig. 5a, b). Interestingly, we found that axial advance rate vs. dedifferentiation probability (Fig. 5b) demonstrates a Michaelis-Menten relationship, determined by regression analysis as axial advance rate = (13.74 *×* *Ω*_DD_)/(0.0262 + *Ω*_DD_) µm/day (with *V*_Max_ = 13.74 and *K*_M_ = 0.0262). Low dedifferentiation rates resulted in lower stem cell density within the DCIS mass (Fig. 5c), and the rate of stem cell population increase was observed to correlate linearly with dedifferentiation probability (Fig. 5d), quantified by best-fitting linear regression as average daily change in stem cell percent density = 0.1839 *×* *Ω*_DD_. A sensitivity score for the results shown in Fig. 5b, d was also determined as we have done previously [27]; this is further discussed in SI and shown in Supplementary Fig. S4.

## Discussion

Our results revealed that, without the dedifferentiation pathway, DCIS behaviors studied in this work (including axial advance rate, proliferation events per day, and axial calcification extent) were most affected by proliferation cycles before differentiation, symmetric probabilities of progenitor cells (progenitor or differentiated daughter), and quiescence thresholds (Table 2, Supplementary Fig. S1). The progenitor population is reduced by proliferation events that give rise to differentiated daughters, terminal differentiation due to hitting the proliferation cycle threshold, and quiescence, which removes proliferative (i.e., progenitor) cells from the population contributing to DCIS growth. Downstream processes that then determine if this population is able to proliferate or not, such as estrogen and FGF thresholds, and cell cycle time before proliferation, showed significant effects on fewer model outputs (a detailed analysis on these effects in our model is presented in [27]). Increased oxygen consumption rates in the DCIS population primarily affected the extent of calcification and total cells lost to hypoxia-induced necrosis, but had minimal effects on DCIS progression rates. This further supports that progression is driven by the leading edge, while hypoxic regions are located at the center of the DCIS mass (this may be observed based on the location of the calcification seen in Fig. 3), and is in good agreement with our previous work [27]. We note that, although our model allows for similar analysis on other potential outputs (e.g., average mitosis cycle length, or detailed analysis of the effects of molecular signaling, which we have previously reported in [27]), we elected to focus on only those that allow for comparison of model output with literature-reported measurements, those pertinent to clinical practice (e.g., mammographic measurement of calcification), or those that are the focus of this study (e.g., growth arrest without dedifferentiation).

Our results reveal that dedifferentiation plasticity is vital for DCIS progression, and that exponential dilution of the bipotent stem-like population ultimately leads to growth arrest as the leading edge of the DCIS mass exhausts its proliferative potential and becomes terminally differentiated (Fig. 4a). We note that the stochasticity of our model allows for stem cell dilution and growth arrest to occur at different times between the two leading edges (e.g., Fig. 3), and axial advance may occur only in one direction for a time after arrest occurs in the opposite leading edge, thereby reducing the total average ductal advance rate and proliferation rate in this case.

Similar to the case without dedifferentiation, progenitor symmetric proliferation probability (*ω*_P_) and quiescence density threshold (*θ*_Q_) were most influential of the examined model outputs when the differentiation pathway was activated (Table 3). Cancer cell oxygen consumption multiplier (*λ*_C_) showed a significant effect on a larger number of examined model outputs when dedifferentiation was active than in the study without dedifferentiation. This is because at longer simulated times, dedifferentiation overcomes growth arrest in the leading edge, allowing for larger DCIS mass and greater total cell counts, and a larger total DCIS volume results in a similarly larger hypoxic (and thus calcified) volume. Interestingly, the dedifferentiation probability (*Ω*_DD_) only showed significant effects on 2/7 total examined model outputs; this is fewer than most parameters examined (Table 3). However, it prevented growth arrest in the leading edge in all simulations performed, thereby demonstrating a necessary role in disease progression. Because lack of dedifferentiation was observed to eventually halt disease progression in all cases examined, this study provides notable evidence that it plays a critical role in the most important clinical aspect: allowing DCIS to overcome growth arrest and facilitating continued disease progression.

The observed trend of lower stem cell density with greater proliferation rates in the global sensitivity analysis (Supplementary Fig. S3) is likely due to exponential expansion in the non-stem (progenitor) cell population, which may double every cell cycle, while dedifferentiation events due to cell plasticity occur at a slower rate. In this study, proliferation rates may be reduced due to unfavorable conditions that are not related to dedifferentiation (such as unsatisfied molecular signaling thresholds), and stem cell density can increase due to continuing dedifferentiation (if *Ω*_DD_ > 0) even in the absence of proliferative events, counterbalancing this effect. However, in our follow-up study wherein only dedifferentiation probabilities were perturbed while all other variables were held at baseline (and thus confounding factors were removed), dedifferentiation rates demonstrated clear relationships between ductal advance rates (Michaelis-Menten) and total stem cell population (linear) (Fig. 5b, d). These revealed that the maximum theoretical axial advance rate per day under the examined model conditions was close to 14 µm/day and half-max advance rates occur at a dedifferentiation probability = 0.0262 per cell cycle (Fig. 5b), while the daily percent change in stem cell density was affected by the dedifferentiation probability according to the linear relation 0.1839 *×* *Ω*_DD_ (Fig. 5d). Reduced stem cell density in the cases of greater cell calcification is likely due to the fact that the center of the DCIS mass (where necrosis leads to hypoxia and calcification) is composed of relatively older cells than at the leading edge. Stochastic dedifferentiation has had more opportunities to occur in these older cells (because they have experienced more cell cycles), and thus stem cell density is likely higher in those regions closer to the site of DCIS initiation. When the region of highest stem cell density is removed from the cell population, the average stem cell density in the remaining population is reduced.

In this study, we implemented dedifferentiation as a purely stochastic event, but it has been reported that dedifferentiation is related to hypoxia in DCIS as well [18, 21]. In upcoming work, we will modify our dedifferentiation mechanism to include a mechanistic hypoxia-based component in order to gain further insights on the mechanistic relationships underlying these phenomena. We are also investigating innate molecular pathways that play natural tumor-suppressing roles in the early stages of DCIS, such as p63 and metalloproteinase-8 [40], which are derived from the mature myoepithelial layer already included in our model. Further, we are collecting in-house data from murine models to identify and calibrate additional key molecular pathways to expand the biological accuracy of the model, and to gain additional insights into molecular effects on the earliest stages of DCIS. Indeed, it has been reported that dedifferentiation is associated with changes in genetic and molecular expression, and also cell conformation, and it is likely that some of these may be distinct from stemness derived directly from a stem-type mother cell [41, 42]. We have now obtained some DCIS RNAseq data, and spatial single cell sequencing is ongoing in our laboratory. These were not included in the current model, and our study is limited in its ability to shed light onto this interesting problem, but it is our hope that this experimental work will reveal specific pathways for inclusion that will allow us to focus future studies on simulated measures of these quantities. We are currently implementing our own mouse model of dedifferentiation in DCIS (and the related but distinct EMT mechanism) based on previously published mouse models [43–45], which we believe will allow additional validation of the results presented and reveal new molecular pathways to be included for study in future model iterations. Ultimately, we hope that this work will help to reveal new insights into potential clinical strategies to optimize emerging cancer stem cell targeted therapeutics, and to predict the risk of DCIS progression into invasive breast cancer. In combination with our upcoming experimental work, we believe that this may lead to identification of one of more pathways that may be targeted to diminish dedifferentiation events in order to halt disease progression in stage zero, and which could help guide drug design in the future. Moreover, we believe that the underlying software we are developing will lead to a tool that enables the study of the effects and interplay of phenotypic hierarchies and molecular signaling in other solid cancer types, leading to new insights into their developmental biology and strategies for therapy.

### Reporting summary

Further information on experimental design is available in the Nature Research Reporting Summary linked to this paper.

## Supplementary information

Supplemental materials

Reporting summary

## Data Availability

All data generated or analyzed during this study are included in this published article and its Supplementary Information files.
