# Supplementary material for: Dedifferentiation-mediated stem cell niche maintenance in early-stage ductal carcinoma in situ progression: insights from a multiscale modeling study
Source: Cell Death Dis. 2022 May 21;13(5):485. doi: 10.1038/s41419-022-04939-x (PMC9124196; doi:10.1038/s41419-022-04939-x)
Supplement: Supplementary file 1 — Supplemental materials [file 41419_2022_4939_MOESM1_ESM.docx]

**Supplementary Information**

**Dedifferentiation-mediated stem cell niche maintenance in early-stage ductal carcinoma in situ progression: insights from a multiscale modeling study**

Joseph D Butner, Prashant Dogra, Caroline Chung, Javier Ruiz-Ramírez, Sara Nizzero, Marija Plodinec, Xiaoxian Li, Ping-Ying Pan, Shu-hsia Chen, Vittorio Cristini, Bulent Ozpolat, George Calin, Zhihui Wang

**Expanded Model Overview**

Within our model, the agent-based model (ABM) discrete scale is hybridized to a continuum scale, wherein Fick’s law of diffusion partial differential equation (PDE) representations of the molecular profiles of interest (specifically oxygen, estrogen, AREG, and FGF) are solved to obtain molecular concentrations across the simulated domain at all time steps, and are explicitly mathematically linked to the ABM scale. This link allows agents to directly modify continuum solutions, accounting for cellular processes such as oxygen metabolism, binding or metabolism of estrogen (e.g., hydroxylation ^1^) and FGF, and ADAM17 mediated cleavage and release of membrane-bound AREG into the extracellular space ^2^. We note that the biological complexities of the mechanisms involved in these molecular interactions are beyond the scope of this study; thus, we have lumped these into “consumption” and “production” terms, which are accounted for by the reaction term *R*(*u*) in the reaction-diffusion equation:

$\frac{du}{dt}=D\nabla^{2}u+R(u)$. (S1)

This allows for solution of all molecular concentrations (*u*) at all time steps (*t*) as a function of respective molecular diffusion constants (*D*). The reaction term *R*(*u*) accounts for both molecular production or consumption *U*(*u*,*x*) and molecular degradation *L*(*u*), such that *R*(*u*) = *U*(*u*,*x*) − *L*(*u*); in all cases, |*U*(*u*,*x*)| >> |*L*(*u*)|. Within the computational domain, all molecules are free to diffuse according to Eq. 1. Boundary conditions (BCs) are Dirichlet for oxygen and estrogen (blood-supplied, and assumed constant), homogeneous Neumann at equilibrium at far-field for AREG (diffuses freely out of the computational domain into the surrounding stroma), and time-dependent Dirichlet for FGF. In the case of FGF, AREG is produced by ER+ agents, and is free to diffuse within the modeled domain until it enters the surrounding stroma and stimulates stromal FGF production (**Fig. 1C**).

PDE solutions were obtained at all time steps via the finite element method (FEM) using Sundance ^3^, a high-level FEM solver, and cell-cell physics were solved using BulletPhysics ^4^. In order to ensure model stability, time step discretization for PDE solutions were finer than for ABM steps, and physics engine solutions were allowed to achieve mechanical relaxation (that is, until agents stop moving due to growth, movement or lysis of their neighbors) between ABM-scale events. We lump all cell molecular consumption to occur at the agent’s center of mass, and at each time step, all agents interpolate the continuum solutions at this location using barycentric interpolation from their nearest surrounding nodes. Agents apply changes to the continuum solutions at their nearest node, determined by Voronoi tessellation around each node, and applied as a Dirac delta function containing the summed contributions per surface area (*a*_i_) or per volume (i.e., *r*_i_) consumption of production values (*λ*_i_) of all agents nearest to each node, e.g.,

$U\left( u,x \right)=\frac{\sum_{i=1}^{n} \lambda_{i}u\int_{-\infty}^{\infty} \delta\left( x-a_{i} \right)\text{d}x}{n}$ . (S2)

Mesh discretization was roughly the same as full-grown agent diameter (**Table S1**) to ensure appropriate scaling between continuum and discrete scales.

Agents in our model are able to under mitosis after completing the previous cell cycle (i.e., proliferation frequency *f*_p_ > τ_P_), provided all conditions for mitosis are satisfied; these are: 1) proliferative or stem phenotype, 2) favorable local molecular concentrations (i.e., above the proliferation threshold; simplified mathematically using the Heaviside function), 3) agent is not quiescent, hypoxic, necrotic, or apoptotic, 4) agent has sufficient room to divide, and 5) agent does not dedifferentiate at this time step. When mitosis occurs, the cytoplasmic volume is divided evenly between the two daughters (note that we do not explicitly model any subcellular or intracellular processes at this time), and the new daughter is placed adjacent to the mother. Daughter phenotypes are selected stochastically based on the hierarchy shown in **Fig. 1C** and the probabilities indicated in **Tables 1, S1**. Daughters grow through a simulated G1 phase until reaching maturity (mature cell radius *r* = 5 μm), and may repeat the mitosis cycle until they either 1) differentiate upon reaching the maximum allowed cell cycles *P*_max_, 2) become quiescent, 3) become necrotic or 4) undergo apoptosis. Cells may become hypoxic when local oxygen concentrations drop below the hypoxia threshold *θ*_H_, and may either return to normoxia if oxygen levels rebound, or will become irreversibly necrotic if hypoxia persists past the necrosis time threshold *τ*_N_. Cells that become necrotic undergo lysis until plasma membrane rupture, after which cytoplasmic contents shrink and become calcified (see **Tables 1, S1** for key parameter values). For a more detailed discussion of these processes and quantification of key parameters, the interested reader may refer to our previous work ^5^.

All computational investigations were performed in a simulated section of mammary duct, represented as a cylinder 1 mm in length with 200 μm inner luminal duct cavity diameter. Before simulation start, cells within the mature mammary epithelial bilayer are seeded, assigned a random phenotype (ER+/−), and the continuum solutions are allowed to stabilize by running the simulation for 10 cell cycles without cancer initiation. Although not directly involved in DCIS, the cells in the mature duct (shown in **Fig. 3**) participate in epithelial and epithelial to stromal signaling, each altering the continuum molecular solutions at their location based on their phenotype (**Fig. 1D**). At the start of DCIS simulation (*t* = 0), a simulated cancer initiation event occurs, wherein 5 adjacent agents in the luminal wall layer ~500 μm from each end of the simulated region are assigned a tumor initiating cell (TIC) phenotype (**Fig. 1B**). These TICs may proliferate indefinitely (immortalized breast cancer lines have been reported to undergo at least 250 cell cycles ^6^) according to the molecular and cell cycle times assigned in each simulation run (**Tables 1, 1**), placing their daughter cells adjacently into the luminal cavity. Daughter phenotypes are determined stochastically, according to values in **Table 1** and the cell hierarchy shown in **Fig. 1C**.

**Mathematical modeling in DCIS and cellular dedifferentiation**

Mathematical modeling of DCIS has revealed valuable insights into the mechanistic underpinnings of DCIS growth and ductal invasion, and many DCIS models have been developed using different approaches ^7^, including agent-based modeling (ABM), continuum, and discrete-continuum, i.e., hybrid modeling approaches. In particular, hybrid modeling of DCIS has been increasingly used to understand the effects of contact inhibition, localized hypoxia, necrosis, calcification ^8^, and acidosis on DCIS architecture ^9^ and the phenotypic evolution of the DCIS cell population ^10^. Macklin *et al*. developed a 2-dimensional hybrid DCIS model, including both an ABM representation of cells and continuum representation of oxygen diffusion, which successfully replicated many of the key aspects of DCIS growth, including cell density, ductal advance rates, and development of hypoxia, necrosis, and calcification, and which was further validated using patient data ^8^. Other notable modeling efforts have yielded both continuum ^11^ and discrete ^9^ representations of DCIS, including ABM models for studying the effects of cell-scale mechanisms on disease progression ^12^.

On the other hand, numerous modeling works have been conducted to study the effects of cell hierarchies in cancer development ^13-18^, and phenotypic plasticity and its relation to cells of origin or CSC population maintenance has received increasing attention in recent modeling studies ^19^. Work by Jilikine and Gutenkust has yielded notable insights into how mutation number and stem vs. progenitor cell population dynamics on dedifferentiation may lead to faster carcinogenesis ^20^, Rhodes and Hillen demonstrated how a survivin-dependent response to radiation therapy may lead to dedifferentiation in NSCLC ^21^, and Zhou and colleagues revealed valuable insights into the adaptive selection of dedifferentiation probabilities ^22^. Other modeling works have shed light onto the relationship between phenotypic plasticity and survival or aggressiveness of the cancer cell population ^23^. Finally, of particular relevance to the study presented herein, modeling of dedifferentiation in breast cancer has helped explain how hypoxia generates dedifferentiation events ^24^, and the roles dedifferentiation may play in breast cancer progression and response to therapeutic intervention ^23,25^.

**Sensitivity Index**

In order to quantify how sensitive these outputs are to perturbations in dedifferentiation probability, we also calculated a sensitivity score as we have done before ^5^, defined as:

$S_{p}^{M}=\frac{{\delta M}/M}{{\delta p}/p}$, (S3)

where *p* is the parameter being varied (in this case, dedifferentiation), *M* is the measured response (axial advance rate and rate of change of stem cell percentage within the viable DCIS population), and *δM* is the change in system response due to the change in parameter value *δp*. Accordingly, the sensitivity of a model output to a known perturbation in input is quantified by $\left| S_{p}^{M} \right|$. Axial advance rates were found to be less sensitive to dedifferentiation probabilities ≤0.2% per cell cycle by our sensitivity index, detailed in Supplemental Materials (Eq. S3), while sensitivity to dedifferentiation probabilities >0.2% per cell cycle was found to remain stably within the $\left| S_{p}^{M} \right|$ = 0.20 – 0.31 range (**Fig. S4A**). Sensitivity score applied to the rate of change of stem cell density within the viable DCIS population was found show notable variability within the range examined, without the emergence of a clear trend (**Fig. S4B**), which is expected based on the linear trend observed in **Fig. 5D**.

**Dedifferentiation rates in other modeling works**

In an experimental work by Chaffer et al. ^26^, a dedifferentiation rate was fit assuming homogenous cell populations, without restrictions on dedifferentiation due to factors such as hypoxia or quiescence to explain the full stem cell population measured. In this work, we chose to use a dedifferentiation probability towards the lower end of the range they report, as the higher reported rates in this work were in cells transformed via instruction of SV40-ER oncogenes, alone or with Ras. Our baseline values (**Table S1**) were validated by stability testing of the stem cell population (not shown), wherein the model was run for long simulated times to confirm that the stem cell population would remain stable within the expected range. Our reported stem cell density at the end of the simulations in the global sensitivity analysis with the dedifferentiation pathway in effect yielded a stem cell density range of 0.50-9.1% (mean 3.6%), thus overlapping experimentally measured values of 4-6% ^26,27^. In the local sensitivity analysis wherein only the dedifferentiation probability was perturbed, stem cell percentages ranged between 0.3-6.3%, and dedifferentiation probabilities ranging between 0.6-0.9% per cell cycle yielded stem cell percentages within the biologically relevant reported range ^26,27^ (corresponding to 4.3% and 5.7% stem cells at the end of the simulation, respectively; **Fig. 5c**).

**Implications of model-imposed permanence in the fully-differentiated phenotype**

It should be noted that in this work, we have made the assumption that differentiation to a non-proliferative state is permanent, only cells with a non-differentiated and non-quiescent phenotype are able to dedifferentiate. However, significant evidence exists that cancer stem cells may originate due to Yamanaka factors (Oct-3/4, Sox2, c-Myc, and Klf4), many of which are oncogenes, that reverse terminally differentiated cells back into pluripotent stem cells ^28^. In this case, dedifferentiation rates would be lower than those tested here, as new stem cells would originate from a larger total cell population (that is, from both differentiated and proliferative populations). Because we observed in our simulations that DCIS progression is driven by proliferation events in the leading edge, where cell density (and thus density-induced quiescence) is lowest and progenitor population is highest (and thus differentiated cell density is the lowest), we do not expect total dedifferentiation events within this regions would be significantly different in this case, and thus this discrepancy is expected to be somewhat reduced. This interesting problem will be further studied in an upcoming work.

As we have shown in a previous study ^5^, cell density-induced quiescence away from the leading edge results in proliferation events primarily occurring within the first few layers of the leading edge, which must be supplemented by a stem cell population to maintain long-term growth. In the model presented here, stem cell distribution in the DCIS mass without dedifferentiation is stochastically determined by the natural course of cell displacement due to proliferation and cell growth, and thus the likelihood of having stem cells in the leading edge is increasingly diluted with time, and can be expected to be fully depleted if enough time were to pass. We note that this could be overcome through other means, such as a directed stem cell migration due to chemotaxis towards higher molecular concentrations ahead of the leading edge, or due to other mechanisms such as progenitor immortalization or higher rates of stem cell self-renewal. However, these were not included in this study.

**References**

1 Yaghjyan, L. & Colditz, G. A. Estrogens in the breast tissue: a systematic review. *Cancer Causes Control* **22**, 529-540, doi:10.1007/s10552-011-9729-4 (2011).

2 Sternlicht, M. D. *et al.* Mammary ductal morphogenesis requires paracrine activation of stromal EGFR via ADAM17-dependent shedding of epithelial amphiregulin. *Development (Cambridge, England)* **132**, 3923-3933, doi:10.1242/dev.01966 (2005).

3 Long, K., Kirby, R. & Waanders, B. v. B. Unified Embedded Parallel Finite Element Computations via Software-Based Fréchet Differentiation. *SIAM J. Sci. Comput.* **32**, 3323-3351, doi:10.1137/09076920x (2010).

4 Coumans, E. in *ACM SIGGRAPH 2015 Courses* 1 (ACM, Los Angeles, California, 2015).

5 Butner, J. D. *et al.* A Multiscale Agent-Based Model of Ductal Carcinoma In Situ. *IEEE transactions on bio-medical engineering* **67**, 1450-1461, doi:10.1109/tbme.2019.2938485 (2020).

6 Wang, J., Hannon, G. J. & Beach, D. H. Risky immortalization by telomerase. *Nature* **405**, 755-756, doi:10.1038/35015674 (2000).

7 Cristini, V., Koay, E. & Wang, Z. *An Introduction to Physical Oncology: How Mechanistic Mathematical Modeling Can Improve Cancer Therapy Outcomes*. (CRC Press, 2017).

8 Macklin, P., Edgerton, M. E., Thompson, A. M. & Cristini, V. Patient-calibrated agent-based modelling of ductal carcinoma in situ (DCIS): from microscopic measurements to macroscopic predictions of clinical progression. *Journal of theoretical biology* **301**, 122-140, doi:10.1016/j.jtbi.2012.02.002 (2012).

9 Boghaert, E., Radisky, D. C. & Nelson, C. M. Lattice-Based Model of Ductal Carcinoma In Situ Suggests Rules for Breast Cancer Progression to an Invasive State. *PLOS Computational Biology* **10**, e1003997, doi:10.1371/journal.pcbi.1003997 (2014).

10 Gatenby, R. A. *et al.* Cellular adaptations to hypoxia and acidosis during somatic evolution of breast cancer. *Br J Cancer* **97**, 646-653, doi:10.1038/sj.bjc.6603922 (2007).

11 Franks, S. J., Byrne, H. M., Mudhar, H. S., Underwood, J. C. & Lewis, C. E. Mathematical modelling of comedo ductal carcinoma in situ of the breast. *Mathematical medicine and biology : a journal of the IMA* **20**, 277-308, doi:10.1093/imammb/20.3.277 (2003).

12 Edgerton, M. E. *et al.* A novel, patient-specific mathematical pathology approach for assessment of surgical volume: application to ductal carcinoma in situ of the breast. *Analytical cellular pathology (Amsterdam)* **34**, 247-263, doi:10.3233/acp-2011-0019 (2011).

13 Youssefpour, H., Li, X., Lander, A. D. & Lowengrub, J. S. Multispecies model of cell lineages and feedback control in solid tumors. *Journal of theoretical biology* **304**, 39-59, doi:10.1016/j.jtbi.2012.02.030 (2012).

14 Enderling, H. *et al.* Paradoxical dependencies of tumor dormancy and progression on basic cell kinetics. *Cancer research* **69**, 8814-8821, doi:10.1158/0008-5472.can-09-2115 (2009).

15 Werner, B. *et al.* The Cancer Stem Cell Fraction in Hierarchically Organized Tumors Can Be Estimated Using Mathematical Modeling and Patient-Specific Treatment Trajectories. *Cancer research* **76**, 1705-1713, doi:10.1158/0008-5472.can-15-2069 (2016).

16 Dingli, D., Traulsen, A. & Michor, F. (A)symmetric stem cell replication and cancer. *PLoS Comput Biol* **3**, e53, doi:10.1371/journal.pcbi.0030053 (2007).

17 Weekes, S. L. *et al.* A multicompartment mathematical model of cancer stem cell-driven tumor growth dynamics. *Bulletin of mathematical biology* **76**, 1762-1782, doi:10.1007/s11538-014-9976-0 (2014).

18 Johnston, M. D., Edwards, C. M., Bodmer, W. F., Maini, P. K. & Chapman, S. J. Mathematical modeling of cell population dynamics in the colonic crypt and in colorectal cancer. *Proceedings of the National Academy of Sciences of the United States of America* **104**, 4008-4013, doi:10.1073/pnas.0611179104 (2007).

19 Jilkine, A. Mathematical Models of Stem Cell Differentiation and Dedifferentiation. *Current Stem Cell Reports* **5**, 66-72, doi:10.1007/s40778-019-00156-z (2019).

20 Jilkine, A. & Gutenkunst, R. N. Effect of dedifferentiation on time to mutation acquisition in stem cell-driven cancers. *PLOS Computational Biology* **10**, e1003481-e1003481, doi:10.1371/journal.pcbi.1003481 (2014).

21 Rhodes, A. & Hillen, T. Mathematical Modeling of the Role of Survivin on Dedifferentiation and Radioresistance in Cancer. *Bulletin of mathematical biology* **78**, 1162-1188, doi:10.1007/s11538-016-0177-x (2016).

22 Zhou, D., Luo, Y., Dingli, D. & Traulsen, A. The invasion of de-differentiating cancer cells into hierarchical tissues. *PLOS Computational Biology* **15**, e1007167, doi:10.1371/journal.pcbi.1007167 (2019).

23 Tonekaboni, S. A. M., Dhawan, A. & Kohandel, M. Mathematical modelling of plasticity and phenotype switching in cancer cell populations. *Mathematical biosciences* **283**, 30-37, doi:10.1016/j.mbs.2016.11.008 (2017).

24 Dhawan, A. *et al.* Mathematical modelling of phenotypic plasticity and conversion to a stem-cell state under hypoxia. *Scientific reports* **6**, 18074, doi:10.1038/srep18074 (2016).

25 Sehl, M. E., Shimada, M., Landeros, A., Lange, K. & Wicha, M. S. Modeling of Cancer Stem Cell State Transitions Predicts Therapeutic Response. *PLoS One* **10**, e0135797-e0135797, doi:10.1371/journal.pone.0135797 (2015).

26 Chaffer, C. L. *et al.* Normal and neoplastic nonstem cells can spontaneously convert to a stem-like state. *Proceedings of the National Academy of Sciences of the United States of America* **108**, 7950-7955, doi:10.1073/pnas.1102454108 (2011).

27 Mani, S. A. *et al.* The epithelial-mesenchymal transition generates cells with properties of stem cells. *Cell* **133**, 704-715, doi:10.1016/j.cell.2008.03.027 (2008).

28 Takahashi, K. & Yamanaka, S. Induction of pluripotent stem cells from mouse embryonic and adult fibroblast cultures by defined factors. *Cell* **126**, 663-676, doi:10.1016/j.cell.2006.07.024 (2006).

29 Washburn, N. R., Weir, M. D. & Yamada, K. M.  Cytokine Dynamics in Three-Dimensional Extracellular Matrices. *Wound Repair and Regeneration* **13**, A28-A48, doi:10.1111/j.1067-1927.2005.130216b.x (2005).

30 Paine, I. *et al.* A Geometrically-Constrained Mathematical Model of Mammary Gland Ductal Elongation Reveals Novel Cellular Dynamics within the Terminal End Bud. *PLOS Computational Biology* **12**, e1004839-e1004839, doi:10.1371/journal.pcbi.1004839 (2016).

31 Carreau, A., El Hafny-Rahbi, B., Matejuk, A., Grillon, C. & Kieda, C. Why is the partial oxygen pressure of human tissues a crucial parameter? Small molecules and hypoxia. *J Cell Mol Med* **15**, 1239-1253, doi:10.1111/j.1582-4934.2011.01258.x (2011).

32 Höckel, M. & Vaupel, P. Biological consequences of tumor hypoxia. *Seminars in oncology* **28**, 36-41 (2001).

33 Steinbach, J. P., Wolburg, H., Klumpp, A., Probst, H. & Weller, M. Hypoxia-induced cell death in human malignant glioma cells: energy deprivation promotes decoupling of mitochondrial cytochrome c release from caspase processing and necrotic cell death. *Cell death and differentiation* **10**, 823-832, doi:10.1038/sj.cdd.4401252 (2003).

34 Grönroos, M. *et al.* Methotrexate induces cell swelling and necrosis in renal tubular cells. *Pediatric blood & cancer* **46**, 624-629, doi:10.1002/pbc.20471 (2006).

35 Wu, L.-Y. *et al.* The anti-necrosis role of hypoxic preconditioning after acute anoxia is mediated by aldose reductase and sorbitol pathway in PC12 cells. *Cell Stress Chaperones* **15**, 387-394, doi:10.1007/s12192-009-0153-6 (2010).

36 Majno, G. & Joris, I. Apoptosis, oncosis, and necrosis. An overview of cell death. *The American journal of pathology* **146**, 3-15 (1995).

37 Cox, R. F. *et al.* Microcalcifications in breast cancer: novel insights into the molecular mechanism and functional consequence of mammary mineralisation. *Br J Cancer* **106**, 525-537, doi:10.1038/bjc.2011.583 (2012).

38 Luby-Phelps, K. Cytoarchitecture and physical properties of cytoplasm: volume, viscosity, diffusion, intracellular surface area. *International review of cytology* **192**, 189-221, doi:10.1016/s0074-7696(08)60527-6 (2000).

39 Sidell, B. D. Intracellular oxygen diffusion: the roles of myoglobin and lipid at cold body temperature. *The Journal of experimental biology* **201**, 1119-1128 (1998).

40 Courant, F. *et al.* Assessment of circulating sex steroid levels in prepubertal and pubertal boys and girls by a novel ultrasensitive gas chromatography-tandem mass spectrometry method. *The Journal of clinical endocrinology and metabolism* **95**, 82-92, doi:10.1210/jc.2009-1140 (2010).

41 Thorne, R. G., Hrabetová, S. & Nicholson, C. Diffusion of epidermal growth factor in rat brain extracellular space measured by integrative optical imaging. *Journal of neurophysiology* **92**, 3471-3481, doi:10.1152/jn.00352.2004 (2004).

42 Wagner, B. A., Venkataraman, S. & Buettner, G. R. The rate of oxygen utilization by cells. *Free Radic Biol Med* **51**, 700-712, doi:10.1016/j.freeradbiomed.2011.05.024 (2011).

**SI Figures**

**Figure S1.**


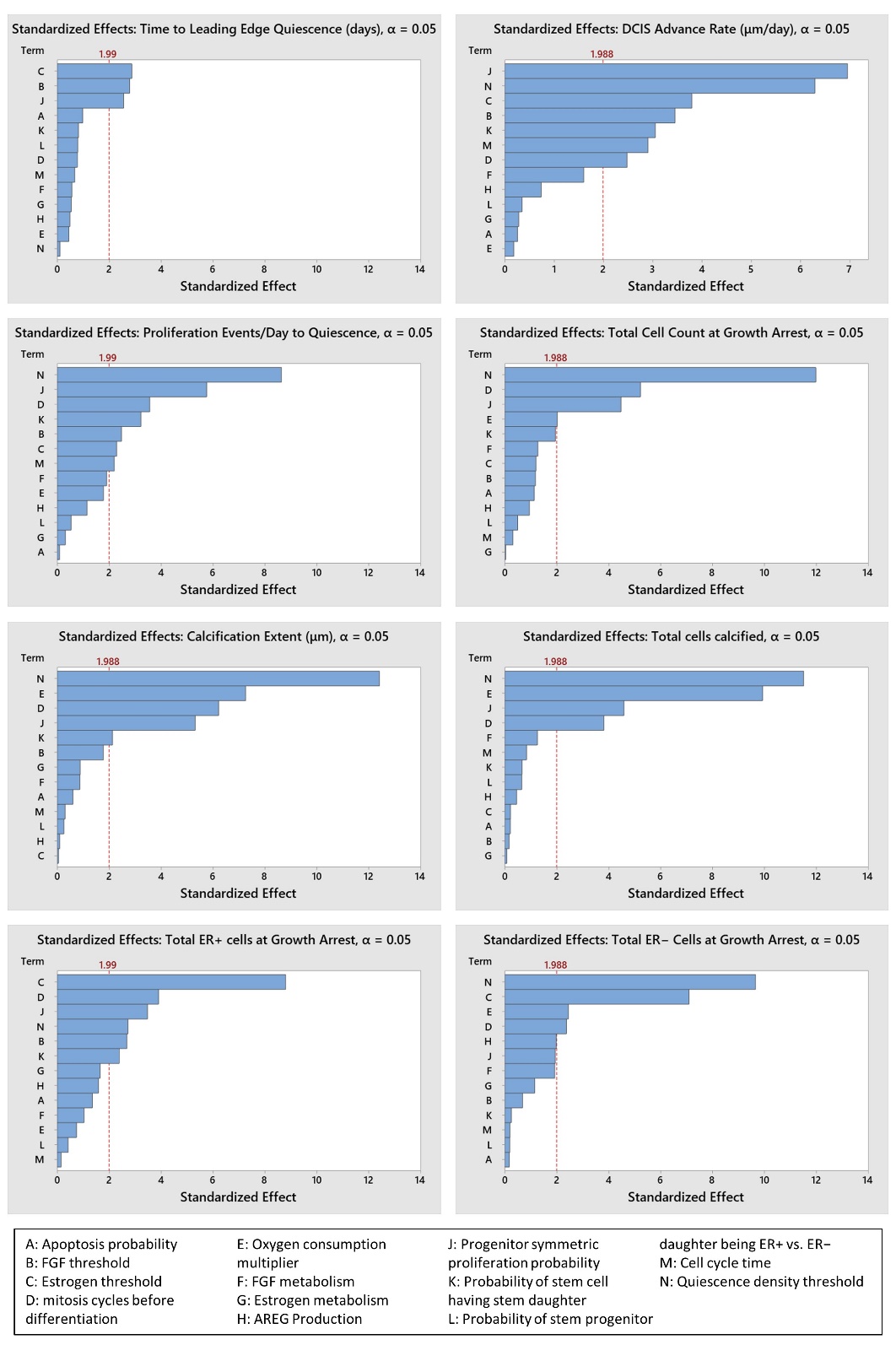


**Fig. S1**: Pareto chart of significant factors on each output examined without dedifferentiation. Plots were generated with Minitab based using multivariate linear regression analysis. Bars that cross the red dashed line are statistically significant by p < 0.05.

**Figure S2.**


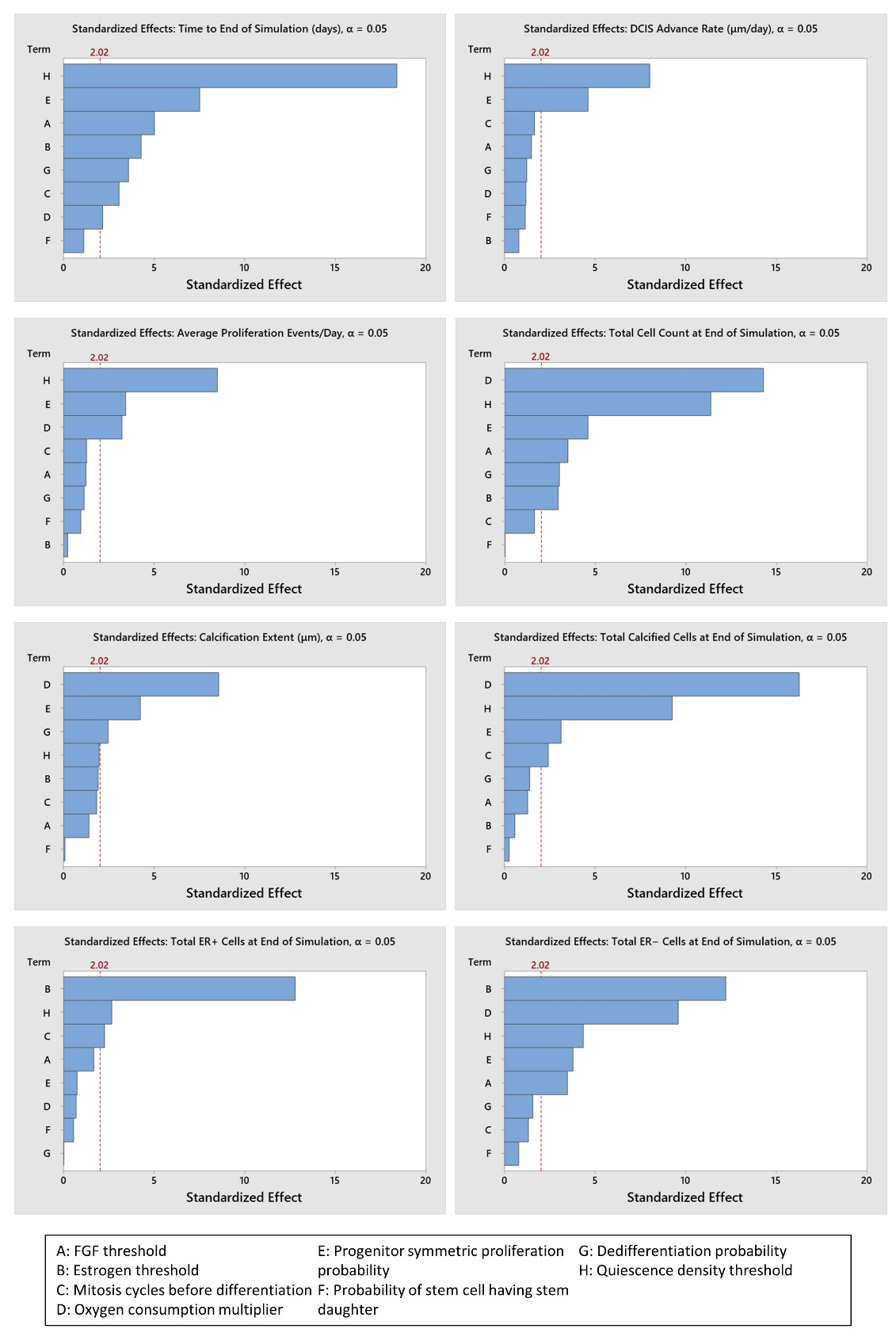


**Fig. S2:** Pareto chart of significant factors on each output examined with dedifferentiation. Plots were generated with Minitab based using multivariate linear regression analysis. Bars that cross the red dashed line are statistically significant by p < 0.05.

**Figure S3.**


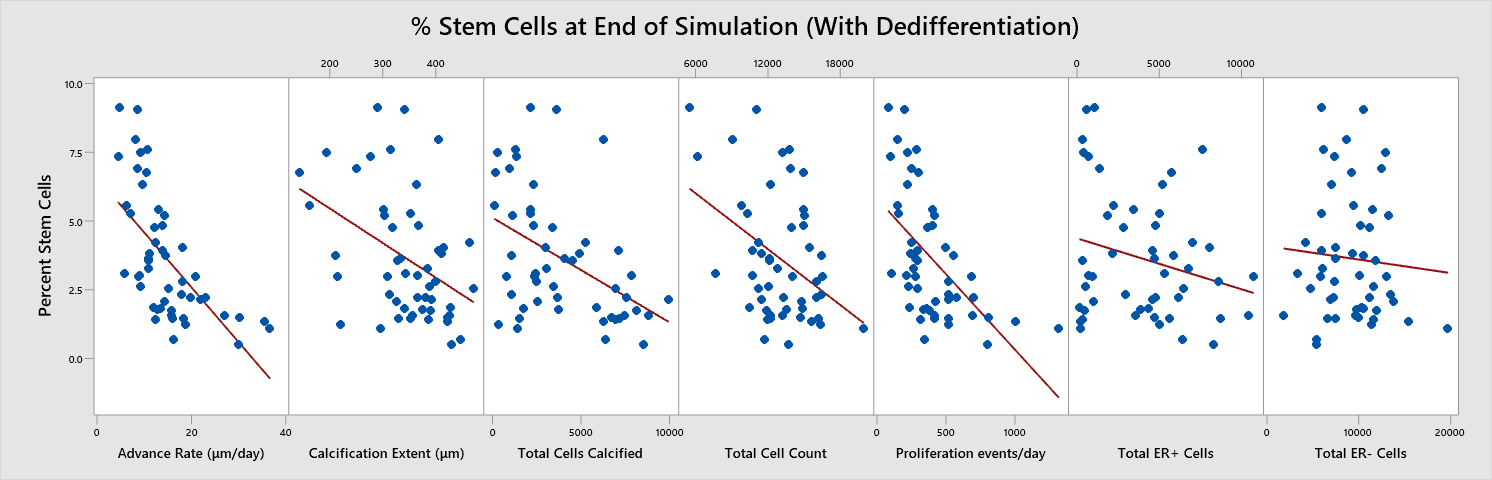


**Fig. S3: Percentage of stem cells in the DCIS cell mass vs. model outputs of interest.** Each point represents the model state at *t* = 96 wall clock hours of model run time from one simulation run in the LHS study (n = 50) of the model parameter space (**Table 1**, main text), with linear regression fits shown in red to show the overall trend. The stochastic dedifferentiation probability was the same for all non-quiescent, progenitor phenotype agents in each run (but different among simulations).

**Figure S4.**


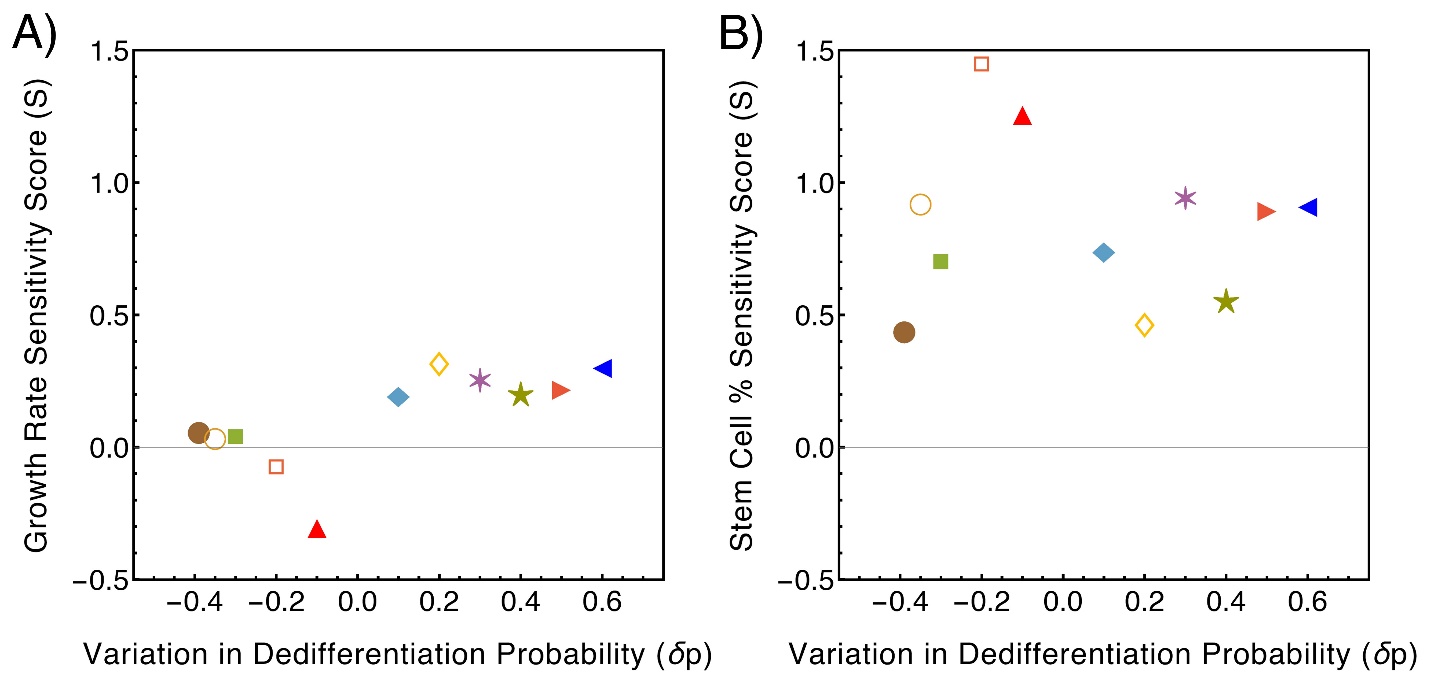


**Fig. S4:** Sensitivity scores (Eq. S3) for dedifferentiation only study for A) growth rates shown in **Fig. 5B** (main text), and B) stem cell % shown in **Fig. 5D** (main text).

**Figure S5.**


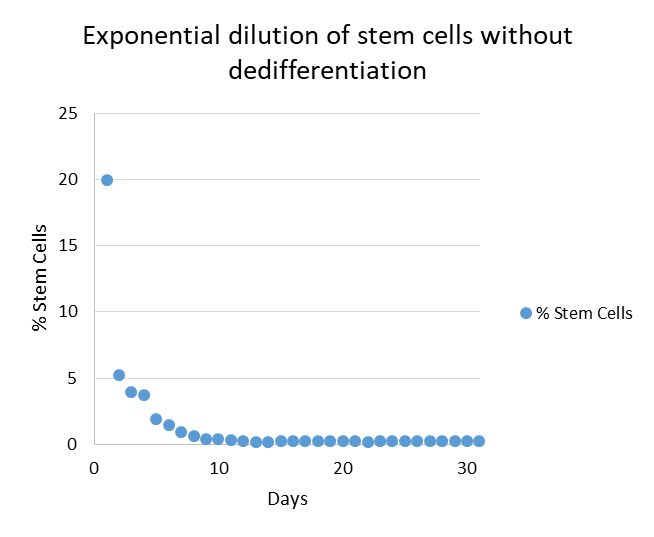


**Fig S5**: Example of stem cell dilution from a simulation without the dedifferentiation pathway active (chosen at random). Parameter values (negative values represent molecular metabolism/uptake): *τ*_P_ = 19.5 hours, *ω*_SC_ = 12.72%, *ω*_P_ = 99.80%, *ω*_SC,+/–_ = 52.00%, *ω*_Apop_ = 0.5337%, *P*_max_ = 14, *Ω*_DD_ = 0.0, *θ*_Q_ = 0.5500, *λ*_C_ = 4.646, *λ*_E_ = -1.030, *λ*_FGF_ = -0.7238, *λ*_AREG_ = 0.09114, *θ*_E_ = 0.6515, *θ*_FGF_ = 0.6948.

**Table S1: Baseline values for model parameters held constant in all global sensitivity studies**. When direct measurements of diffusion constants were unavailable from the literature, values were interpolated either from structurally similar molecules^†^ or molecules with similar molecular weights^††^. ^†††^Diffusion constant of bFGF has been directly measured via fluorescence correlation spectroscopy ^29^ in various collagen substrates ranging ~1.3-3.2×10^−7^ cm^2^s^−1^; taking the higher end of this range we approximate it using the same value for AREG for simplicity. *Dedifferentiation was not perturbed in the first phase of the study, but was perturbed in the second part.

| **Parameter** | **Symbol** | **Baseline Value** | **Reference** |
| --- | --- | --- | --- |
| **Discrete (ABM) parameters** | | | |
| Mature mammary cell radius | *r* | 5 μm | ^30^ |
| Hypoxia threshold | *θ*_H_ | 1/3 normoxia | ^31,32^ |
| Hypoxia time to necrosis | *τ*_N_ | 12 hours | ^33^ |
| Lysis volume increase due to swelling | *V*_L_ | 100% | ^34,35^ |
| Lysis time | *τ*_L_ | 6 hours | ^36^ |
| Time to calcification | *t*_C_ | 14 days | ^37^ |
| Calcified volume (% of pre-lysis cell volume) | *θ*_Q_ | 30% pre-lysis volume | ^38^ |
| **Continuum parameters** | | | |
| Blood oxygen concentration (constant) | $C_{O_{2}}$ | 100 mmHg | ^31^ |
| Oxygen diffusion constant | D_oxygen_ | 2.5 × 10^-6^ cm^2^ s^−1^ | ^39^ |
| Blood Estrogen concentration (constant) | *C*_Estrogen_ | 70.3 pMol L^-1^ | ^40^ |
| Estrogen diffusion constant | *D*_estrogen_ | 2.45 × 10^-6^ cm^2^ s^−1^ | ^†^ |
| AREG diffusion constant | *D*_AREG_ | 3.18 × 10^-7^ cm^2^ s^−1^ | ^41††^ |
| FGF diffusion constant | *D*_FGF_ | 3.18 × 10^-7^ cm^2^ s^−1^ | ^29†††^ |
| **Hybrid (ABM ↔ continuum) parameters** | | | |
| Healthy cell oxygen consumption rate | *λ*_E_ | 45 × 10^-18^ Mol cell^−1^ s^−1^ | ^42^ |

**Table S2: Effects of dedifferentiation on DCIS advance rate and stem cell population.** Linear regression analysis in the form of *y* = *mx* + *b* was performed to quantify the rate constant *m*, representing average axial advance rate per day and the average rate of change of stem cell percentage within the viable DCIS cell population shown in **Fig. 5A** (axial advance rate) and **Fig. 5C** (stem cell percentage) after time *t* ≥ 15 days.

| Probability (%/cell cycle) | DCIS advance rate (μm/day) [*R*^2^] | Stem cell population (% change/day) [*R*^2^] |
| --- | --- | --- |
| 0.01 | 3.911 [0.985] | 5.000×10^−3^ [0.718] |
| 0.05 | 9.310 [0.988] | 1.145×10^−2^ [0.870] |
| 0.1 | 10.660 [0.989] | 2.709×10^−2^ [0.928] |
| 0.2 | 12.855 [0.996] | 3.491×10^−2^ [0.926] |
| 0.3 | 13.217 [0.999] | 5.670×10^−2^ [0.982] |
| 0.4 | 11.755 [0.991] | 8.257×10^−2^ [0.967] |
| 0.5 | 12.469 [0.992] | 9.972×10^−2^ [0.968] |
| 0.6 | 13.272 [0.998] | 9.865×10^−2^ [0.921] |
| 0.7 | 13.100 [0.997] | 1.436×10^−1^ [0.970] |
| 0.8 | 13.321 [0.998] | 1.164×10^−1^ [0.902] |
| 0.9 | 14.164 [0.998] | 1.623×10^−1^ [0.929] |
| 1.0 | 14.667 [0.999] | 1.817×10^−1^ [0.986] |
